# A novel anti-CD19 monoclonal antibody (GBR 401) with high killing activity against B cell malignancies

**DOI:** 10.1186/1756-8722-7-33

**Published:** 2014-04-14

**Authors:** Caroline S Breton, Aimable Nahimana, Dominique Aubry, Julie Macoin, Pierre Moretti, Martin Bertschinger, Samuel Hou, Michel A Duchosal, Jonathan Back

**Affiliations:** 1 Service and Central Laboratory of Hematology, University Hospital of Lausanne, Rue du Bugnon 46, 1011- CHUV, Lausanne, Switzerland; 2 Glenmark Pharmaceuticals S.A, Chemin de la Combeta 5, 2300 La Chaux de-Fonds, Switzerland

**Keywords:** B cell malignancies, GBR 401, Anti-CD19 monoclonal antibody, ADCC, Therapeutic antibody

## Abstract

**Background:**

CD19 is a B cell lineage specific surface receptor whose broad expression, from pro-B cells to early plasma cells, makes it an attractive target for the immunotherapy of B cell malignancies. In this study we present the generation of a novel humanized anti-CD19 monoclonal antibody (mAb), GBR 401, and investigate its therapeutic potential on human B cell malignancies.

**Methods:**

GBR 401 was partially defucosylated in order to enhance its cytotoxic function. We analyzed the *in vitro* depleting effects of GBR 401 against B cell lines and primary malignant B cells from patients in the presence or in absence of purified NK cells isolated from healthy donors. *In vivo,* the antibody dependent cellular cytotoxicity (ADCC) efficacy of GBR 401 was assessed in a B cell depletion model consisting of SCID mice injected with healthy human donor PBMC, and a malignant B cell depletion model where SCID mice are xenografted with both primary human B-CLL tumors and heterologous human NK cells. Furthermore, the anti-tumor activity of GBR 401 was also evaluated in a xenochimeric mouse model of human Burkitt lymphoma using mice xenografted intravenously with Raji cells. Pharmacological inhibition tests were used to characterize the mechanism of the cell death induced by GBR 401.

**Results:**

GBR 401 exerts a potent *in vitro* and *in vivo* cytotoxic activity against primary samples from patients representing various B-cell malignancies. GBR 401 elicits a markedly higher level of ADCC on primary malignant B cells when compared to fucosylated similar mAb and to Rituximab, the current anti-CD20 mAb standard immunotherapeutic treatment for B cell malignancies, showing killing at 500 times lower concentrations. Of interest, GBR 401 also exhibits a potent direct killing effect in different malignant B cell lines that involves homotypic aggregation mediated by actin relocalization.

**Conclusion:**

These results contribute to consolidate clinical interest in developing GBR 401 for treatment of hematopoietic B cell malignancies, particularly for patients refractory to anti-CD20 mAb therapies.

## Background

The last decade has seen an increased interest in the use of antibody-based therapeutic approaches for cancer treatment with clear efficacy and safety profiles [1-3]. In this context, anti-CD20 monoclonal antibodies (mAbs), such as the first in line Rituximab (RTX), have been used to target human B cell malignancies, improving the treatment of this group of diseases [4,5]. Anti-CD20 mAbs exert their antitumor activities through various modes of cell death induction including antibody dependent cellular cytotoxicity (ADCC) [6], complement-dependent cytotoxicity (CDC) [7] and direct induction of cell death [4]. However, despite this success, some patients do not respond to this targeted mAb therapy. Some of the main molecular mechanisms underlying the resistance to anti-CD20 mAbs are (a) low expression levels of CD20 (such as in chronic lymphocytic leukemia-CLL) [8]; (b) down regulation of CD20 expression during the treatment [9,10]; (c) expression of CD20 transcript variants lacking the RTX epitope sequence [11] and (d) complement depletion associated with the loss of component C2 [12]. Developing novel and potent mAbs to circumvent such drug resistances is of great importance.

An interesting development is to target CD19, a specific B cell marker, expressed early during pre-B cell ontogeny and until terminal differentiation into early plasma cells, with a potential efficacy on a large panel of B cell malignancies [13]. Indeed, the majority of B cell lineage malignancies (more than 90%) express CD19, including notably non-Hodgkin’s lymphoma, CLL and acute lymphoblastic leukemia (ALL) [14,15]. Finally, tumor B cells that have lost the expression of CD20 after anti-CD20 mAb therapy, maintain the expression of CD19. Given its biology and broad expression pattern, CD19 represents an attractive chemotherapeutic target to treat B cell malignancies. Consequently, efforts are now being deployed to develop novel anti-CD19 mAbs to treat various hematological malignancies and emerging results from preclinical studies are encouraging [16-19].

In the past few years, the knowledge of how the Fc fragment of antibodies can trigger cytotoxic mechanisms has improved substantially. In particular, glycosylation analyses have identified fucose residues as negatively influencing the binding of the Fc to FcγRIIIa. This receptor is expressed by natural killer (NK) cells, neutrophils and monocytes and is known to trigger ADCC. Antibodies bearing low levels of fucose residues have been shown to display enhanced ADCC potential [20,21].

Here we describe GBR 401, a partially defucosylated humanized mAb targeting the domain II of CD19 and derived from the parental mouse anti human CD19 antibody clone FMC63 [19]. Both *in vitro* and *in vivo* data showed that GBR 401 was highly effective at depleting human malignant B cells mainly via ADCC. It also exhibited a direct killing effect on human B cell malignancies. Finally, benchmarking done against RTX, demonstrated a remarkably superior killing capacity of GBR 401. Our preclinical results suggest GBR 401 to be an efficacious therapeutic agent for human B lymphoma and leukemia and warrant further clinical studies of GBR 401 in these diseases.

## Results

### GBR 401 is a partially defucosylated mAb

**Figure 1:**
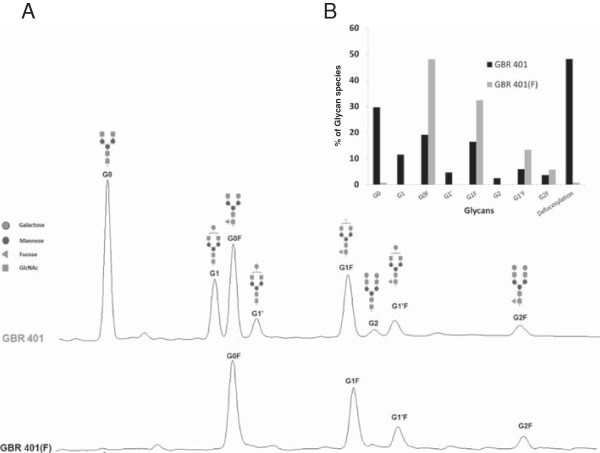
**Fucosylated and non-fucosylated complex N-glycans analysis for GBR 401 and GBR 401(F). A**/ Fucosylated and non-fucosylated complex N-glycans associated with GBR 401 and GBR 401(F) antibodies analyzed by CE. **B**/ Diagram of quantitative data with the total level of defucoylation defined as the sum of G0, G1, G1’ and G2.

GBR 401 is a mAb with enhanced affinity for FcγRIIIa due to its low fucose content. The humanization, binding characteristics and engineering performed to produce GBR 401 are described in Skegro et al. (manuscript in preparation). GBR 401 is produced in a recombinant CHO cell line allowing the expression of mAbs with a reduced level of α1-6 fucose linked to the N-acetylglucosamines in the N-glycan core. The glycosylation of GBR 401 can be seen by HPLC run (Figure 1) and is compared to its fully fucosylated parent GBR 401(F) antibody. Whereas GBR 401(F) shows a normal CHO glycosylation profile with biantennary complex N-oligosaccharides G0F, G1F, G1F’ and G2F, GBR 401 shows a high level of defucosylated glycans G0, G1, G1’ and G2 (Figure 1A). The overall defucosylation level of GBR 401 reaches approximately 50% versus <1% for GBR 401(F) (Figure 1B).

### GBR 401 exhibits a potent *in vitro* ADCC activity on malignant B cells

**Figure 2:**
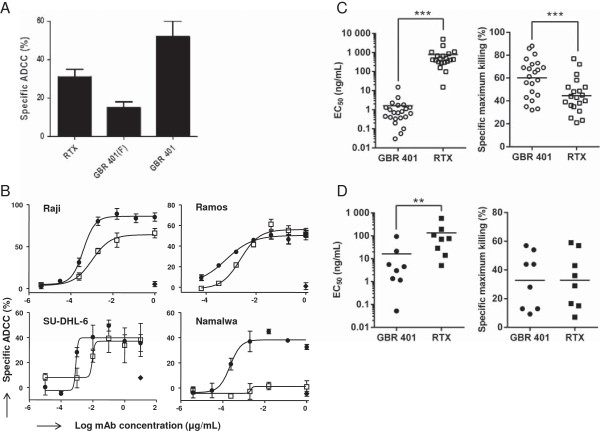
**B leukemia cells are sensitive to GBR 401 mediated ADCC *in vitro*. A**/ Raji cells (Burkitt’s lymphomas, CD19+ CD20+), were incubated during 4 hr with 0.5 μg/mL of mAbs in the presence of purified NK cells from random donors (effector/target ratio of 5). Target cell death was measured by LDH release. Specific ADCC (%) was calculated by the formula: (Sample release – spontaneous release)/(Max release – spontaneous release) x100. The graph shows the average specific ADCC (%) ± SD of triplicates obtained with one NK cell donor representative of several different donors. The Table 1 presents the data summary for all donors. GBR 401(F) is the fully fucosylated variant of GBR 401. **B**/ cell lines Raji, Ramos (Burkitt’s lymphomas, CD19+ CD20+), Namalwa (Burkitt’s lymphoma, CD19+ CD20low) and SU-DHL-6 (Human diffuse histiocytic lymphoma, CD19+ CD20+) were incubated during 4 hr with various concentration of mAbs in the presence of purified NK cells from random donors (effector/target ratio of 5). Target cell death was measured as in **(A)**. The graphs show the average specific ADCC (%) ± SD of triplicates. Each graph shows a representative result obtained from at least four different NK cell donors. The Table 1 presents the data summary for all donors. **C + D**/ PBMC from patients with CLL (n = 13) **(C)** or B cell lymphomas (n = 4) **(D)** were incubated with peripheral blood NK cells (effectors/target ratio of 1) from two different healthy volunteer donors with various concentration of mAbs (0.005 ng/mL - 5 μg/mL) for 24 hr. HER was used as control mAb. The malignant B cells were stained for cell death by 7AAD and analyzed by flow cytometry. Figures show EC_50_ values and maximum killing percentage of specific ADCC for each NK donor and leukemia sample combination.

Since NK cell-mediated ADCC is important for the activity of many mAbs [16,22-24], we first determined the ADCC activity of GBR 401 in the Burkitt’s lymphoma cell line Raji, in comparison with GBR 401(F). In agreement with its low fucose content, GBR 401 displayed a markedly superior ADCC activity compared to the fully fucosylated variant (Figure 2A and Table 1).

**Table 1:** Summary of mean ABC (antibody binding capacity) values and ADCC activities of GBR 401 and RTX on primary B malignancies and B cell lines

**Target cells**	**Antigenic CD19 sites**	**Antigenic CD20 sites**	**EC**_**50 **_**GBR 401 (μg/ml)**	**EC**_**50 **_**RTX (μg/ml)**	**EC**_**50 **_**GBR 401 (F) (μg/ml)**	**Average max killing GBR 401 (%)**	**Average max killing RTX (%)**	**Average max killing GBR 401 (F) (%)**
Raji	57 100 (11 000)	100 400 (13 200)	0.0012	0.012^#^	0.08^#^	80	60	30^#^
Ramos	50 700 (6700)	160 600 (29 700)	0.0009	0.016		60	60	
SU-DHL-6	30 000 (4800)	822 400 (41 000)	0.0008	0.0045		50	40	
Namalwa	37 900 (4500)	1 700 (1200)	0.002	NA		60	15	
B-CLL	10 520 (4140)	3 230 (2000)	0.0016 (0.003)	0.8*** (1.1)		60.04 (16.9)	44.54*** (15.1)	
Lymphoma			0.015	0.15**		32	32	

The table displays ADCC values obtained with at least 4 different NK cell donors for Raji, Ramos, SU-DHL-6 and Namalwa cells. The EC_50_ values are given in μg/mL (SD). The average max killing is given in % (SD). Antigenic CD19 and CD20 sites values are mean (SD). NA, Not applicable. Stars or # indicate statistical significance compared to GBR 401 value (*< .05, **< .01, ***< .001 by student t test, ^#^< .05 by Wilcoxon ranked test).

We next evaluated the ADCC potential of GBR 401, in comparison with RTX, the standard mAb for lymphoma therapy and herein used as a positive control and reference. When we tested the ADCC activity on cell lines where CD19 and CD20 were highly expressed (Raji, Ramos and SU-DHL-6, Table 1), GBR 401 and RTX showed a comparable maximum killing efficacy (Figure 2B). However, Namalwa cells that express low levels of CD20 (Table 1) responded solely to GBR 401. Despite similar maximum killing efficacies, and substantially higher levels of CD20 compared to CD19 (Table 1), GBR 401 demonstrated an overall 6-10 fold lower EC50 compared to RTX on various malignant B cells that include Raji, Ramos and SU-DHL-6 cells indicating a better ADCC activity at low doses (Table 1 and Figure 2B). Of interest, we also investigated whether GBR 401 was able to kill primary malignant cells. To this end, peripheral blood mononuclear cells (PBMC) from patients diagnosed with CLL and other types of lymphomas (mantle cell lymphoma, Waldenström lymphoma, hairy cell leukemia-variant and post-transplant lymphoproliferative disorder) were treated with increasing concentrations of GBR 401 or RTX in the presence of NK cells purified from two healthy donors. As shown in Figure 2C, GBR 401 showed a strikingly potent *in vitro* ADCC activity against B-CLL cells. Indeed, it decreased by 500 fold (*P* < .001) the mean EC_50_ values (ranging between 0.056 and 15.2 ng/mL) compared with RTX (15-5000 ng/mL). The maximum killing efficacy of GBR 401 was also significantly superior (*P* < .001) to that of RTX (Figure 2C). With primary lymphoma samples, GBR 401 reduced EC_50_ values (*P* < .01, 0.05-93 ng/mL) compared with RTX (5-580 ng/mL) (Figure 2D), even though there was no difference in their maximum killing efficacy. These results can partially be explained by the relatively low expression of CD20 in our CLL samples (Table 1 and Additional file 1: Figure S1), which is in agreement with literature data [16,25]. However, CD19 expressions in CLL samples are very low when compared with our cell lines and are not vastly different from those of CD20 (Table 1 and Additional file 1: Figure S1) [25]. Similarly, CD20 levels on lymphoma cells have been reported to be higher than CD19 levels [25]. These observations indicate that the better activity of GBR 401 on CLL and lymphoma samples cannot be solely explained by a bias in CD19 levels but rather by a better cytotoxic activity.

Taken together, these data demonstrate that GBR 401 displays potent ADCC activity against various cell lines and primary CLL and lymphoma samples. Interestingly, GBR 401 shows far superior ADCC potential over RTX in settings where CD20 expression is reduced as is seen in some CLL samples and in Namalwa cells. This indicates that GBR 401 could be an interesting alternative therapeutic agent in such conditions where RTX is poorly effective.

### GBR 401 is highly efficient at depleting B cells in xenografted SCID mice

**Figure 3:**
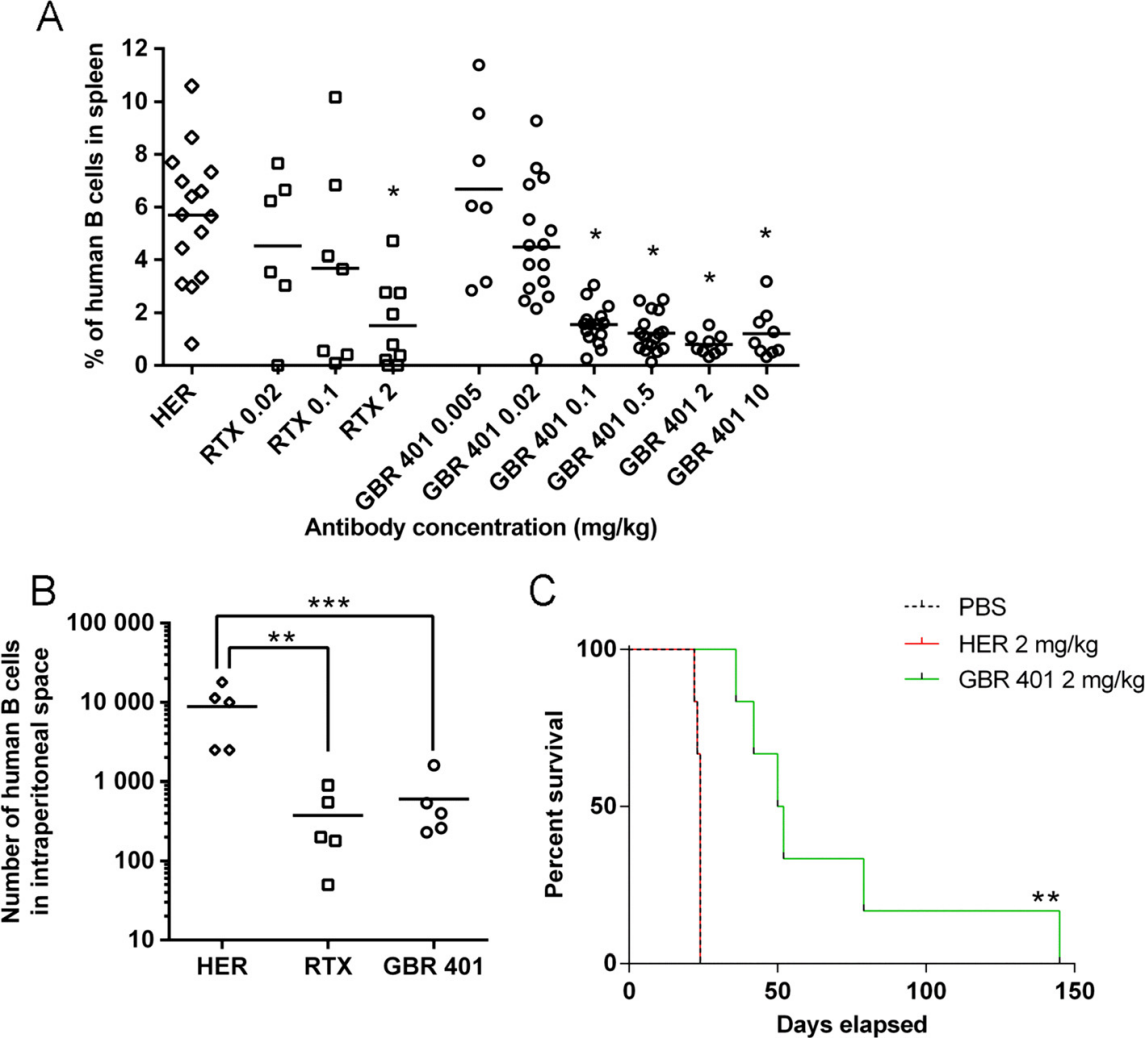
**GBR 401 can deplete B cells in xenografted SCID mice. A**/ SCID mice were irradiated, reconstituted with human PBMCs and treated with mAbs. Depletion of human B cells was monitored 4 days after antibody treatment by quantifying the proportion of total human B cells in the spleen. The graph shows pooled data from two independent studies. HER isotype control was dosed at 10 mg/kg and 2 mg/kg. **B**/ Patient derived CLL (n = 5) (15×10^6^) were injected i.p. in SCID mice simultaneously with purified NK cells from healthy donors (effectors/target ratio of 6) and mAb treatment (2 mg/kg). The mice were sacrificed seven days later and cell death was analyzed on peritoneal CLL cells (CD45 + CD5 + CD3-). The graph shows the absolute alive B-CLL cell number found in the peritoneal cavity. **C**/ Groups of 6 SCID mice were injected i.v. with 2 × 10^5^ Raji cells (day 0). Mice were treated with mAbs (GBR 401, HER, 2 mg/kg) or PBS alone at day three, seven and ten, and monitored daily and sacrificed when hind leg paralysis or signs of illness appeared. All control groups died within 24 days after Raji injection while GBR 401 increased median survival by 2.1 fold (*P* < .01).

The promising *in vitro* ADCC potential of GBR 401 prompted us to investigate its *in vivo* efficacy. Given the lack of cross reactivity of GBR 401 to relevant non-human species (unpublished data) it was not possible to test the toxicological effect of GBR 401 in a classical animal model. Human tumor cells xenografted into immunodeficient mice are commonly used to assess the efficacy of oncology drugs on human target cells. We therefore evaluated the efficacy of GBR 401 in a SCID mouse model irradiated to deplete murine NK cells (to increase human cell engraftment), and repopulated with human PBMCs. These latter cells provide both the human B cell targets as well as human effector cells (NK cells and monocytes), and hence this model is expected to display all the potential depletion mechanisms that could occur in humans (Figure 3A). GBR 401 demonstrated a clear dose response for B cell depletion, with an EC_50_ value of 0.03 mg/kg and a maximum efficacy from 2 mg/kg with 84% reduction of the B cell population 4 days after mAb treatment. In comparison, the dose at which RTX lost efficacy (0.1 mg/kg) was at least 5 fold higher than GBR 401 (0.02 mg/kg) despite their similar efficacies at the dose of 2 mg/kg.

The efficacy of GBR 401 in killing normal B cells is of clinical interest in the potential to deplete primary malignant B cells from patients. To test this issue, SCID mice were co-injected intra-peritoneally with B-CLL from patients and heterologous NK cells from healthy donors, at a ratio of one NK cell for six CLL cells (Figure 3B). This NK cell supplementation aimed at mimicking the presence of tissue NK cells that would be present in the organs of CLL patients, but that are at very low levels in PBMC. Compared to mice treated with the isotype control (herceptin®, HER), mice that received GBR 401 at a single dose of 2 mg/kg displayed a dramatic and significant reduction (92%) of B-CLL cells in the peritoneal cavity after seven days (*P* < .001). At the same dose RTX exhibited similar CLL depletion activity to GBR 401 (*P* < .01). We next evaluated the anti-tumor activity of GBR 401 in a disseminated lymphoma model in which human Raji cells expressing high levels of CD19 were injected intravenously into SCID mice and GBR 401 administered as described in the “Materials and Methods”. In this model, Raji cells invade all mouse organs including the central nervous system and mimic diseases such as disseminated Burkitt’s lymphoma and ALL. GBR 401 treatment significantly prolonged mice survival by 2.1 fold (*P* < .01), compared with animals injected with vehicle or control mAb HER (Figure 3C). The median survival time was 51 days for the GBR 401-treated mice compared with 24 days for the control antibody HER or PBS-treated ones.

Collectively, these data demonstrate the capacity of GBR 401 to deplete malignant B cells and prolong mice survival in multiple SCID mouse models.

### CDC is not involved in GBR 401 mediated cell death

To further characterize the cell death mechanisms underlying the antitumor activity of GBR 401, we investigated whether CDC could be involved in GBR 401-mediated cell death. CDC is another potential mechanism of action of mAbs and plays a major role in the efficacy of RTX [26,27]. As expected, the addition of complement to the *in vitro* cultures allowed RTX to significantly increase cell death, whereas GBR 401 was unable to trigger CDC on Raji cells (Additional file 2: Figure S2). However, this inability to trigger CDC is not a characteristic specific to GBR 401 as numerous other anti-CD19 cytotoxic mAbs in development are reported to lack CDC activity [17,28-30].

### GBR 401 induces direct cell death and inhibits the clonogenic capacity of malignant B cells

**Figure 4:**
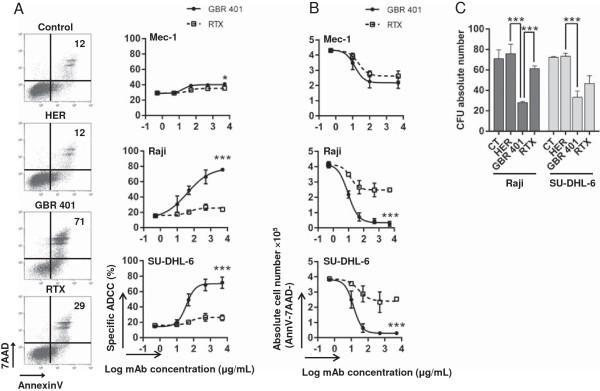
**GBR 401 induces homotypic adhesion and cell death *in vitro.*** Raji, SU-DHL-6 and Mec-1 cell lines were incubated with mAbs (0.1 ng/mL – 5 μg/mL) for 2 hours. **A**/ Cell death was measured in flow cytometry by annexinV/7AAD. Flow cytometry panels are representative of at least three independent experiments (mAbs at a concentration of 1 μg/mL) and numbers indicate the percentage of dead cells (annexinV + 7AAD-, annexinV + 7AAD+, annexinV-7AAD+). The graphs show the percentage of dead cell +/- SD of three independent experiments. **B**/ Cell depletion after mAb incubation was assessed with CountBright™ Absolute Counting Beads. The graphs represent the absolute alive cell number +/- SD (annexinV-7AAD-) counted by flow cytometry experiment. **C**/ The clonogenic capacities of Raji and SU-DH-L6 cell lines were assessed with CFU assays. 500 cells were treated with the mAbs (1 μg/mL) for 2 hours and plated on methylcellulose medium containing proliferation factors. Fourteen days later, the colonies were counted under an inverted microscope. The graph shows the mean +/- SD of three independent experiments.

Direct induction of cell death is a process described for mAbs engaging CD19 or CD20 [18,29,31-34]. The induction of cell death by GBR 401 on various human B lymphoma cells was therefore evaluated *in vitro* using flow cytometry and annexinV/7AAD staining. A short time-exposure (2 hrs) of GBR 401 to Mec-1, Raji and SU-DHL-6 cell lines resulted in a rapid increase in the proportion of late apoptotic/necrotic dead (annexinV + 7AAD+/annexin-7AAD+) or early apoptotic dead (annexinV + 7AAD-) cells with maximum killing effects of 70-80% when compared to the isotype control HER (Figure 4A). The direct killing effect on these malignant B cells was variable; Mec-1 being less to moderately sensitive, whereas Raji and SU-DHL-6 were highly sensitive to GBR 401 treatment, suggesting a cell based specificity for direct cell death induction. The effect of RTX on these three cell lines was very weak, indicating a selective mechanism of rapid cell death induction on malignant lymphoid B cells by GBR 401 (Figure 4A). To provide additional evidence for direct cytotoxicity of GBR 401 on malignant B cells, the absolute B cell number was quantified after mAb treatments. In agreement with annexinV and 7AAD staining data, GBR 401 was much more potent than RTX at reducing the number of malignant B cells after drug exposition (Figure 4B). To further demonstrate the tumor growth inhibitory effects of GBR 401 (in comparison with RTX) we evaluated the capacity of malignant B cells treated with these mAbs to form colonies on methylcellulose semi-solid medium. As shown in Figure 4C, GBR 401 substantially decreased the CFU activity (*P* < .001) compared to RTX on Raji and SU-DHL-6 cells after 14 days.

Collectively, these data support a direct cytotoxicity potential for GBR 401 and highlight its potent killing effects on malignant B cells.

### GBR 401-mediated cell death requires actin reorganization

**Figure 5:**
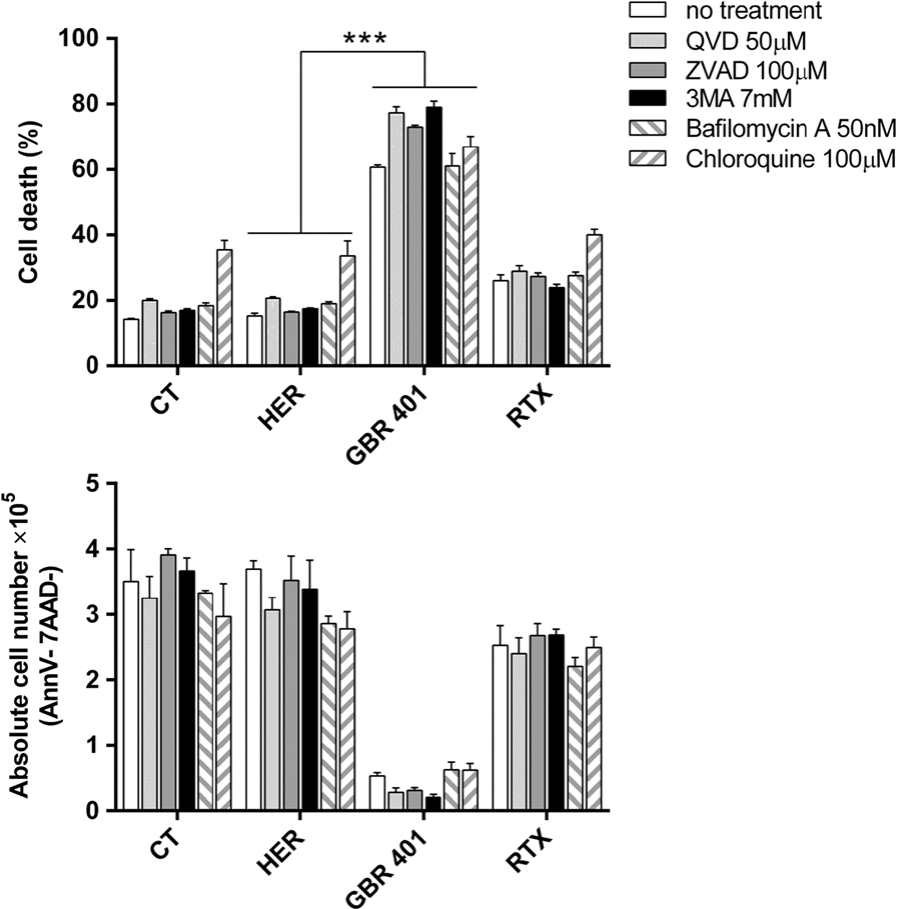
**GBR401 does not trigger apoptosis and autophagy.** Raji cells were pre-incubated with inhibitors of caspases (QVD, ZVAD), of lysosomal proteases (Chloroquine, Bafilomycin A), or of autophagosome formation (3MA) and were treated with mAbs (1 μg/mL) for 2 hours. Absolute alive cell numbers +/- SD and cell death +/- SD were then measured by CountBright™ Absolute Counting Beads and annexinV/7AAD flow cytometry staining.

To elucidate the molecular mechanisms underlying GBR 401-mediated cell death, we first sought to identify the cell death program involved in this process. GBR 401-treated malignant B cells underwent phosphatidylserine externalization, as evidenced by annexinV staining, suggesting apoptotic cell death. To determine whether caspase activation was involved, Raji cells were pre-incubated with two broad spectrum caspase inhibitors (QVD and zVAD), prior to GBR 401 exposition. As shown in Figure 5, none of the inhibitors attenuated GBR 401-induced cell death, supporting a caspase-independent pathway. We next investigated whether autophagy could be implicated in this process. Autophagy is normally a cytoprotective mechanism, but in some stressed conditions, it has the capacity to induce cell death [35]. To test this, Raji cells were pre-incubated with different autophagic inhibitors including a blocker of autophagosome formation (3MA) and inhibitors of lysosomes (Bafilomycin A and Chloroquine), and subsequently treated with GBR 401. None of these inhibitors could prevent GBR 401-killing effects (Figure 5), ruling out any link between GBR 401-induced cytotoxicity and lysosome permeabilization, a cell feature most often associated with mAb-induced cell death [36,37]. We also excluded the involvement of reactive oxygen species (ROS) production, massive internalization of CD19, mRNA transcription, and new protein synthesis in GBR 401-induced direct cytotoxicity (Additional file 3: Figure S3).

**Figure 6:**
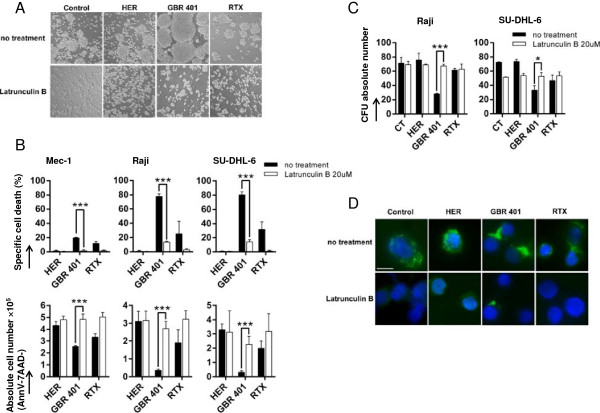
**Cell death induced by GBR401 is dependent on homotypic adhesion and on actin microfilament reorganization.** Mec-1, Raji and SU-DHL-6 cells were pre-incubated with the inhibitor of actin polymerization latrunculin B and treated with mAbs (1 μg/mL) during 2 hours. **A**/ Homotypic adhesion was assessed by light microscopy (original magnification x20). Raji cells are shown as a representative example. **B**/ Cell death and absolute alive cell number were analyzed by annexinV/7AAD flow cytometry staining. **C**/ The clonogenic capacities of Raji and SU-DHL-6 cells were assessed with CFU assays. **D**/ Raji cells were sedimented onto microscope slides. After fixation with 4% paraformaldehyde in PBS, cells were permeabilized, stained with Phalloïdin-A488 and analyzed by fluorescent microscopy (scale bar: 10 μm). All the measurements were realized in triplicates and the graphs represent mean values +/- SD.

Homotypic aggregation is a particular cell process that has been reported to be associated with mAb-induced cell death [33,34,38]. We then assessed whether homotypic aggregation could be involved in this killing process. To address this issue, Raji cells were treated with GBR 401 or RTX, and subsequently cell adhesion was visualized using light microscopy. As shown in Figure 6A (no treatment condition), GBR 401 induced a rapid and strong homotypic aggregation on Raji cells whereas RTX treatment generated little aggregation. This observation suggests a correlation between homotypic aggregation and GBR 401-induced cell death. Actin reorganization has been reported to play a key role in homotypic aggregation and cell death [39,40]. To examine whether actin reorganization is required in GBR 401-mediated direct cytotoxicity, various malignant B cells were treated with GBR 401 or RTX in the presence or absence of latrunculin B, an inhibitor of actin polymerization. As expected, latrunculin B fully prevented the homotypic aggregation induced by these mAbs (Figure 6A). Importantly, the blockage of homotypic cell adhesion by latrunculin B was associated with a loss of direct cytotoxicity induced by GBR 401 and RTX, as revealed by annexinV/7AAD staining (Figure 6B). Similarly, the absolute cell number and the CFU counts were equivalent between the HER control treated cells and GBR 401-treated ones in the presence of latrunculin B (Figure 6B and C). To further clarify the involvement of actin cytoskeleton reorganization in cell death induction after GBR 401 exposure, immuno-fluorescence labeling of actin was performed on treated-Raji cells. As shown in Figure 6D, we observed an important redistribution of actin at the cell-cell contact zones in GBR 401-treated cells. In contrast, RTX was not able to induce this massive relocalization. In accordance with the data mentioned above, latrunculin B prevented the polymerization of actin and its relocalization induced by GBR 401 (Figure 6D), indicating that actin integrity and reorganization are required for GBR 401-induced cell death.

Altogether these results indicate that the ligation of GBR 401 on B cells induces a homotypic cell aggregation through actin cytoskeleton mobilization and relocalization at the cell-cell synapse, which leads to the induction of non canonical apoptotic pathways.

## Discussion

B cell depletion therapy using mAbs alone or in combination with chemotherapeutic drugs has greatly improved the treatment of various hematological malignancies and prolonged overall patient survival [41]. Anti-CD20 mAbs, notably RTX, however, may induce resistance in patients [42] and alternative strategies are needed. GBR 401, a novel glyco-engineered and humanized anti-CD19 mAb, another B-cell-specific cell surface antigen, is a potential complementary to anti-B cell mAbs. Indeed, we demonstrate in the present study that GBR 401 exerts a potent *in vitro* and *in vivo* cytotoxic activity on malignant cells derived from cell lines and from patients diagnosed with various B cell malignancies.

ADCC is a major mechanism of cell death triggered by mAbs. Moreover, several studies have demonstrated an enhancement of ADCC activity of mAbs due to fucose depletion in the Fc region N-linked glycans [43-47], resulting in a higher binding affinity toward FcγRIIIa. In agreement, GBR 401 exhibited an increased ADCC activity on primary malignant B cells. Strikingly, we observed a substantial difference in the ADCC efficacy of GBR 401 between B-CLL and lymphoma samples (Figures 2C and D) even though lymphoma and CLL samples are reported to express similar amounts of CD19 [25]. In addition we did not observe a correlation between the killing efficiency of GBR 401 and CD19 levels on CLL samples (Additional file 2: Figure S2). These observations suggest that the global efficacy of GBR 401 may depend on the intrinsic characteristics of the malignant B cell rather than, or in addition to, the level of CD19 expression. This is in agreement with the fact that GBR 401 is able to trigger ADCC at very low concentrations, when engaging a small number of CD19 molecules. This property to induce B cell depletion by engaging a very low number of receptors on target cells might be crucial to achieve efficacy in patients where the number of effector cells can be limiting. Additionally, GBR 401 efficiently depleted malignant CD19+ B cells expressing low levels of CD20 (such as primary B-CLL and Namalwa cells) which are poorly affected by anti-CD20 mAb treatment. This was reflected in a stark 500 fold better EC50 for GBR 401 compared to RTX on primary CLL cells. Interestingly, while the MEDI-551 anti-CD19 antibody also showed better killing on a limited number of primary CLL samples, the difference with RTX was not as marked as with GBR 401 [16]. We could not compare killing efficacies of GBR 401 to other anti-CD19 or defucosylated anti-CD20 mAb currently in development, since none of them were commercially available at the time of realization of our experiments. The comparison of GBR 401 and defucosylated anti-CD20 antibodies will provide essential data on the efficiencies of CD19 and CD20 targeting in various B-cell malignancies. Nevertheless our data support the high efficacy of targeting CD19.

These observations underline that patients with B-CLL could benefit from GBR 401 treatment and also strengthen the rationale of developing anti-CD19 mAb therapy as an alternative or complementary strategy to circumvent the resistance to, or the failure of, anti-CD20 mAb therapies associated with low levels of CD20 expression, downregulation or expression of CD20 transcript variants [48].

GBR 401 does not trigger CDC on malignant B cells, a common feature of anti-CD19 mAbs in development [28-30]. However, we found by various readouts, including flow cytometry using annexinV/7AAD staining, cell count number and clonogenic assay, that GBR 401 is highly effective at inducing direct cell death (i.e. without the requirement of Fc-mediated mechanisms) in malignant B cells. GBR 401-induced direct cell death was confirmed in a xenochimeric SCID mouse model, in which GBR 401 treatment significantly prolonged overall survival in mice bearing human lymphoma when compared with xenochimeric mice injected with isotypic Ig. As expected in this experiment, RTX also prolonged xenochimeric mouse survival even to a greater extent than GBR 401 (data not shown). This model lacks the human effector cell component and thereby did not evoke the full ADCC potential of GBR 401, while RTX still benefited from CDC and to a greater extent, compared to GBR 401, from ADCC triggered by mouse effector cells (our unpublished data). However, the contribution of murine macrophages in the protective effect of GBR 401 could not be excluded. Indeed, in previous studies using other anti-CD19 mAbs with increased binding affinity toward FcγRIIIa, it was clearly demonstrated that murine macrophages (but not NK or neutrophils) were involved in the *in vivo* B cell depletion [28]. The contribution of macrophages or direct cell death in the GBR 401 protective effect in mice bearing human lymphoma remains to be further clarified.

Our study also provides some insights into the molecular mechanisms involved in the direct cell death evoked by GBR 401. We showed that GBR 401-induced direct cell death did not involve autophagy, caspase activation or lysosomal permeabilization but strongly correlated with homotypic aggregation mediated by the relocalization of actin toward the cell-cell interface. These observations are consistent with previous studies, showing that the actin cytoskeleton was playing a key role in the cell death induced by various mAbs [34,38,40,49].

In conclusion, we demonstrated that GBR 401, a defucosylated humanized anti-CD19 mAb, can induce ADCC and direct-cell death on both normal B cells and malignant B cell lines but more importantly on primary B cell malignancies. All studies were benchmarked against RTX, the current anti-CD20 mAb standard immunotherapeutic treatment for B cell malignancies, and demonstrated high killing capacities for GBR 401. This study contributes to consolidate clinical interest in developing anti-CD19 mAbs for the treatment of hematopoietic B cell malignancies, particularly for patients resistant or refractory to anti-CD20 mAb therapy or relapsing after such treatment.

## Materials & methods

### Cell lines, primary cells and culture conditions

Raji, Ramos, Namalwa (human Burkitt’s lymphoma cell lines), SU-DHL-6 (human diffuse histiocytic lymphoma cell line) and Mec-1 (human B-CLL cell line) were obtained from the German Collection of Microorganisms and Cell Cultures (DSMZ, Germany) or the American Type Culture collection (ATCC, USA). NK cells and peripheral blood mononuclear cells (PBMC) were isolated from fresh buffy coat layers from healthy donors (Lausanne Blood Transfusion Centre). PBMC were purified after Ficoll gradient centrifugation (Histopaque®-1077 Sigma-Aldrich). To obtain NK cells, PBMC were enriched in CD56+ cells using MACS separation with MicroBeads (Miltenyi Biotec). Purity of NK cells (between 80% and 95%) was assessed by flow cytometry.

Bone marrow or PBMC cells from 22 patients with CLL or B cell lymphomas were collected after human ethical committee approval from our institution. Cells were cultivated in RPMI (Gibco) with 10% heat-inactivated fetal calf serum (FCS, Gibco), 100 U/mL penicillin and 100 μg/mL streptomycin (Bioconcept) at 37°C, 5% CO2. Study protocol (reference 295/11) was approved by the ethics committee at the University of Lausanne.

### Mice

SCID mice (8 weeks old) were purchased from Iffa Credo (L’Arbresle, France) and maintained under specific pathogen-free conditions within the animal facilities at the University Hospital of Lausanne. The mice were handled in accordance with institutional regulations after approval of the animal ethic committee of the University of Lausanne. Manipulations were performed under a laminar flow hood under sterile conditions.

### Monoclonal antibodies and carbohydrate analysis

GBR 401 was obtained from Glenmark Pharmaceuticals S.A. The analysis of the carbohydrates associated with GBR 401 and with the fucosylated form GBR 401(F) was performed by capillary electrophoresis (CE) using a PA 800 analysis system with a 488 nm Laser Module and a LIF detector (Beckman Coulter Inc). The separation was achieved with an N-CHO coated capillary, all buffer and labeling reagents were obtained from the Carbohydrate Labeling and Analysis Kit from Beckman Coulter.

The isotype-matched control herceptin® (HER) and rituximab® (RTX) were the clinical products.

### Flow cytometry

Anti hCD45-FITC, hCD3-ECD, hCD5-PECy7, hCD20-ECD and hCD19-PECy7 were purchased from Immunotech. Anti hCD56-PE was purchased from Becton Dickinson Biosciences Pharmingen. FACS analysis was performed on a Beckman Coulter Cytomics FC500 flow cytometer and data were analyzed with the Kaluza Version 1.1 software (Beckman Coulter). Expression levels of CD19 and CD20 were determined using QIFIKIT flow cytometry (Dako) according to the manufacturer instruction. Mouse anti hCD19 (clone HIB19, eBioscience) and hCD20 (clone B9E9, Becton Dickinson Biosciences Pharmingen) were used as primary antibodies.

### Cell death induction and inhibition

Cells were incubated 2 hours with mAbs (HER, GBR 401 or RTX, 1 μg/mL). Cell death inhibitors were pre-incubated 1 hour before adding mAbs. 3-Methyladenine (3-MA, 10 mM), Q-VD-OPh (QVD, 50 μM), Chloroquine (100 μM) and Bafilomycin A (50nM) were purchased from Sigma-Aldrich. ZVAD-fmk (ZVAD, 100 μM) was purchased from BachemAG. Latrunculin B (20 μM) was purchased from Calbiochem. Cell death was assessed using AnnexinV-FITC (eBioscience) and 7AAD (Beckman Coulter) staining. Absolute cell number was characterized with CountBright™ absolute counting beads (Molecular Probes).

### Detection of cellular and mitochondrial reactive oxygen species (ROS)

The intracellular reactive oxygen species H_2_O_2_ level was measured using 5-(and-6)-carboxy-2',7'-dichlorodihydrofluorescein diacetate (Carboxy-H_2_DCFDA, Molecular Probes). Briefly, the cell-permeable molecule becomes green-fluorescent when oxidized with intracellular H_2_O_2_. Cells were incubated during 30min at 37°C with 7μM Carboxy-H2DCFDA and green fluorescence was measured on flow cytometry. The mitochondrial superoxides were measured with MitoSOX™ Red Mitochondrial Superoxide Indicator (Molecular Probes). The molecule selectively targets mitochondria, and becomes red-fluorescent when oxidized with superoxide specifically. Cells were incubated during 30min at 37°C with 5μM MitoSOX in PBS with Ca/Mg and red fluorescence was measured on flow cytometry. The ROS scavenger Tiron (5mM), the protein synthesis inhibitor Cycloheximide (50μM), the mRNA transcription inhibitor Actinomycine D (5μM) and the endocytosis inhibitor Dynasore (50μM) were purchased from Sigma-Aldrich (St Louis, MO).

### ADCC experiments

Target cells were stained with mAbs (HER, GBR 401 or RTX) for 15 minutes (concentrations between 0.005 ng/mL and 5 μg/mL). NK cells, activated by incubation with IL-2 (100 U/mL) during 16 hours, were added at a ratio NK cells/target cells of 1/1 (experiments on primary cells) or 5/1 (experiments on cell lines) and were incubated for 4-24 hours at 37°C. ADCC efficacy was analyzed by cytometry (experiments on primary cells) by quantifying the absolute number of living target cells after incubation. CLL cells were identified as CD3-CD5+, B lymphoma cells as CD19 + CD20+ cells, NK cells were CD3-CD56+ and dead cells were 7AAD+. ADCC was also determined by LDH release (experiments on cell lines) using the CytoTox96 kit (Promega) according to manufacturer’s instructions. Specific ADCC was determined by subtracting specific effect induced by mAbs to specific effect induced by NK cells and control mAb HER.

### CDC experiments

Endogenous CD19-expressing tumor cells (Raji) were harvested and washed once with 1× PBS. Baby rabbit complement (Harlan) was reconstituted with sterile water according to the manufacturer’s instructions. Cells were incubated with 2.5% baby rabbit complement in culture medium for 3 hr at 37°C. The cellular mortality was quantified with the Cytotox 96 non radioactive cytotoxicity assay (Promega), according to the manufacturer’s instructions. This assay quantifies the LDH release by dead cells via a colorimetric enzymatic reaction. OD readings translate to the quantity of LDH released and therefore cell mortality. Percent cytotoxicity was calculated as follows. First, the background signal measured in wells containing only medium was subtracted to all other values. The formula was: Specific CDC (%) = [(SpR-SR)/(MR – SR)] ×100, where SpR corresponds to the LDH release measured in a sample. SR corresponds to the spontaneous LDH release measured for target cells alone. MR corresponds to the maximum release measured for target cells submitted to 3 freeze/thaw cycles.

### *In vivo* B cell depletion experiments

For primary CLL cell depletion, NK cells (ratio NK cells/target cells of 1/6) and mAbs (2 mg/kg) were injected simultaneously into the peritonea of SCID mice. Seven days after injection, animals were euthanized and treatment efficacy was analyzed by determining the number of human B cells (CD45 + CD19 + CD20+) in the peritoneal cavity of mice. For the healthy donor PBMC depletion, SCID mice were sub-lethally irradiated at day 0, depleted for murine NK cells using the TMbeta-1 antibody (mCD122 antigen, BioXCell, USA) on day 1 and 8, and injected with 30 × 10^6^ human PBMC on day 3. Animals were treated with mAbs at day 14 (single bolus injection) and sacrificed at day 18. The spleens were analyzed by flow cytometry to determine the proportion of human B cells in human CD45+ population. Total human B cells were identified as the sum of CD19+ CD20-, CD19+ CD20+ and CD19- CD20+ populations. For the xenograft model of Raji, cells (2 × 10^5^ cells) were injected intravenously in SCID mice. Animals were divided into three groups that received GBR 401 (2 mg/kg, n = 6), HER (2 mg/kg, n = 6), or saline (n = 6) at days 3, 7 and 10. Animals were monitored daily for signs of illness and killed if hind limb paralysis was noted.

### Light microscopy and immunofluorescence imaging

Raji cells were pre-incubated 1 hour with latrunculin B (20 μM) and treated 2 hours at 37°C with mAbs (1 μg/mL) in flat-bottomed 24-well plates. Cell aggregation was assessed in light microscopy using the Leica DM IL inverted microscope at the original magnification ×20. For immunofluorescence, cells were fixed in paraformaldehyde 4%, sedimented onto SuperFrost slides (Menzel-Glaser), permeabilized in 0.1% Triton X-100 and stained with Alexa488-Phalloidin (Molecular Probes). Slides were mounted in FluorSave reagent (Calbiochem), and acquired on an Axioplan 2 fluorescent microscope (Zeiss).

### Clonogenic assays

Raji and SU-DHL-6 cell lines were treated 2 hours with mAbs (1 μg/mL) and evaluated for their capacity to form colonies. 500 treated cells were plated in 1 mL semi-solid methylcellulose medium (Methocult GF + H4435, StemCell Technologies). Colonies (>50 cells) were scored under an inverted microscope after 14 days at 37°C with 5% CO_2_.

### Statistics

Data were expressed as mean plus or minus standard deviation (SD). Student’s t-tests or Wilcoxon ranked tests were performed to determine differences between samples. GraphPad Prism version 6.02 (GraphPad Software, San Diego, CA) was used for statistical analysis. *P* values inferior to .05 were considered statistically significant (* < .05, ** < .01, *** < .001).

## Competing interests

C.S.B., A.N., D.A. and M.A.D. declare no competing financial interest. J.M., S.H., P.M., M.B. and J.B. are employees by Glenmark Pharmaceuticals SA.

## Authors’ contributions

Contribution: JB, SH and MAD initiated the study; CSB, AN, JB and MAD designed the research and coordinated the project; CSB, DA, PM, MB and JM performed experiments; all authors analyzed data; JB and SH provided essential material; CSB, AN, PM and JB wrote the paper. All authors edited the paper, and approved the final manuscript.

## Supplementary Material

Additional file 1: Figure S1 In vitro ADCC efficacy for GBR 401 is not related to antigen expression. A/ CD19 and CD20 site numbers of patient derived CLL cells (n = 5) were calculated according to Qifikit protocol as described in materials & methods. B/ PBMC from patients with CLL (n = 5) were incubated with peripheral blood NK cells (effectors/target ratio of 1) from two different healthy volunteer donors with various concentration of mAbs (0.005 ng/mL - 5 μg/mL) for 24 hr. HER was used as control mAb. The malignant B cells were stained for cell death by 7AAD and analyzed by flow cytometry. Figures show the average specific ADCC (%) ± SD of three representative CLL samples with one NK cell donor. C/ Figures show EC50 values and maximum killing percentage of specific ADCC versus the antigenic site number of CD19 or CD20 for all patient derived CLL cells tested (n = 5). Click here for file

Additional file 2: Figure S2 GBR 401 does not trigger complement-dependent cytotoxicity Standard CDC assays were performed incubating Raji cells with mAbs (1 μg/mL) and 2.5% baby rabbit complement. The isotype control was Herceptin. The graph shows the mean specific cytotoxicity (%) +/- SD of triplicates. Click here for file

Additional file 3: Figure S3 GBR 401 cell death is not induced by ROS production, CD19 internalization or by mRNA and protein syntheses. A/ Raji cells were pre-incubated with the ROS scavenger Tiron and were treated for 2 hours with mAbs (1 μg/mL). Cell death (annexinV + 7AAD+/-), intracellular H2O2 (Carboxy- H2DCFDA) and mitochondrial superoxide (Mitosox) were assessed by flow cytometry. B/ Raji cells were pre-incubated with inhibitors of endocytosis (Dynasore), mRNA transcription (Actinomycin D) or protein synthesis (Cycloheximide) and were treated for 2 hours with mAbs (1 μg/mL). Cell death (annexinV + 7AAD+/-) was assessed by flow cytometry. Click here for file
