# Supplementary figures and images for: A novel anti-CD19 monoclonal antibody (GBR 401) with high killing activity against B cell malignancies
Source: J Hematol Oncol. 2014 Apr 14;7:33. doi: 10.1186/1756-8722-7-33 (PMC4021825; doi:10.1186/1756-8722-7-33)

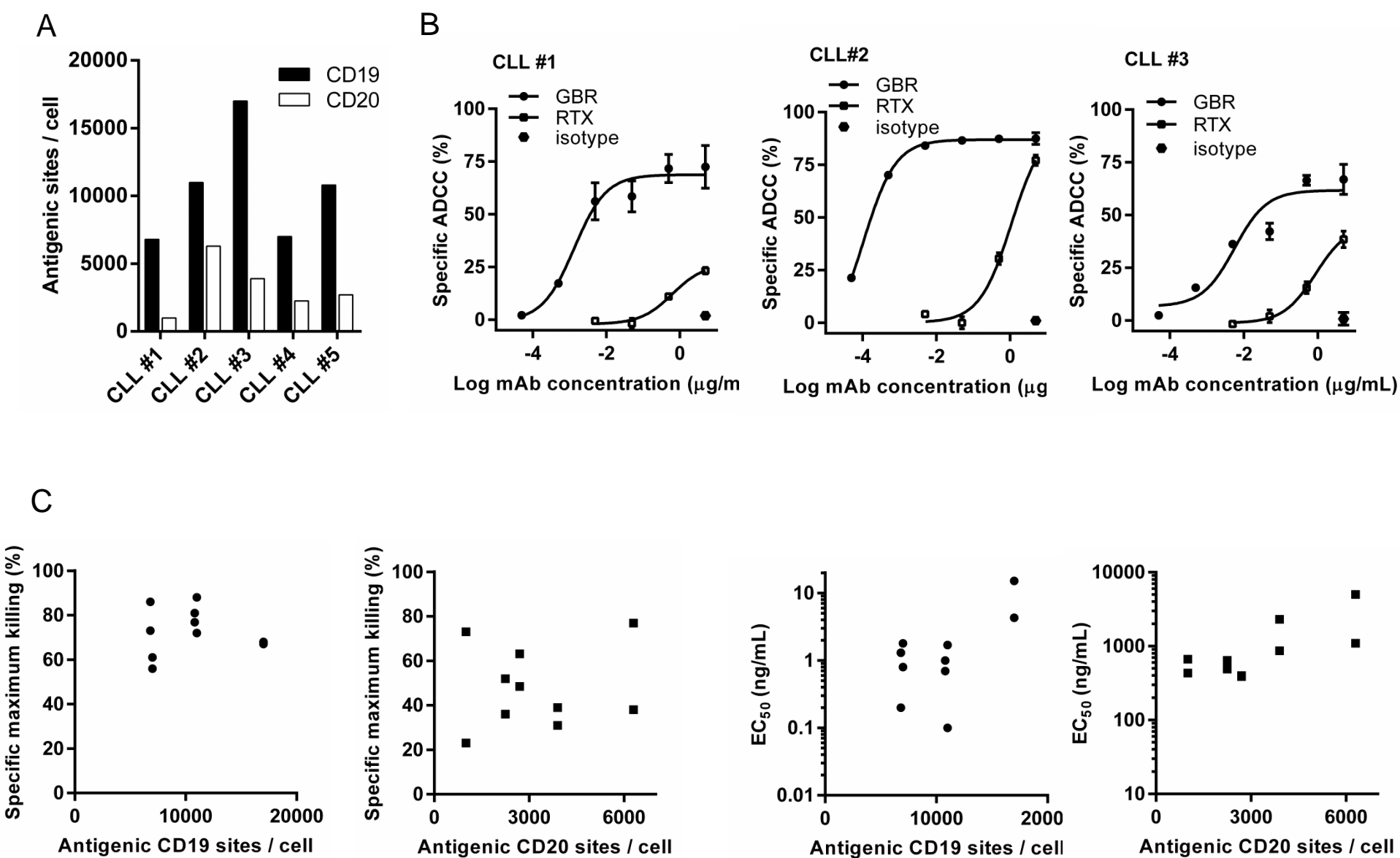

**Figure S1**

Supplement: Additional file 1: Figure S1 — In vitro ADCC efficacy for GBR 401 is not related to antigen expression. A/ CD19 and CD20 site numbers of patient derived CLL cells (n = 5) were calculated according to Qifikit protocol as described in materials & methods. B/ PBMC from patients with CLL (n = 5) were incubated with peripheral blood NK cells (effectors/target ratio of 1) from two different healthy volunteer donors with various concentration of mAbs (0.005 ng/mL - 5 μg/mL) for 24 hr. HER was used as control mAb. The malignant B cells were stained for cell death by 7AAD and analyzed by flow cytometry. Figures show the average specific ADCC (%) ± SD of three representative CLL samples with one NK cell donor. C/ Figures show EC50 values and maximum killing percentage of specific ADCC versus the antigenic site number of CD19 or CD20 for all patient derived CLL cells tested (n = 5). [file 1756-8722-7-33-S1.pdf]

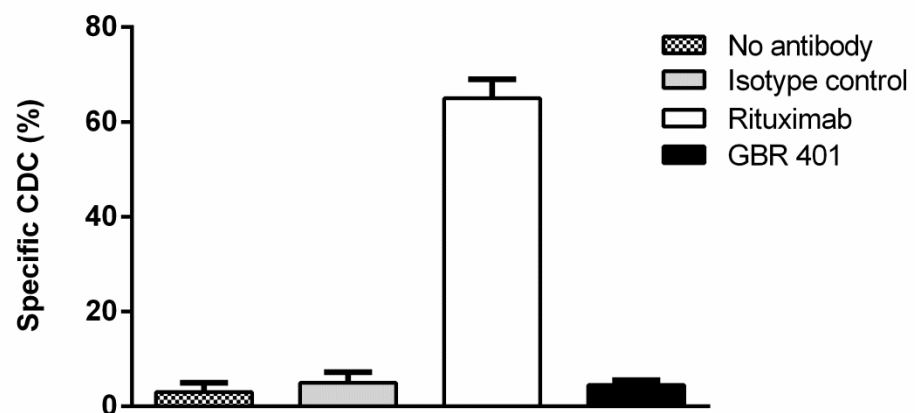

**Figure S2**

Supplement: Additional file 2: Figure S2 — GBR 401 does not trigger complement-dependent cytotoxicity Standard CDC assays were performed incubating Raji cells with mAbs (1 μg/mL) and 2.5% baby rabbit complement. The isotype control was Herceptin. The graph shows the mean specific cytotoxicity (%) +/- SD of triplicates. [file 1756-8722-7-33-S2.pdf]

A

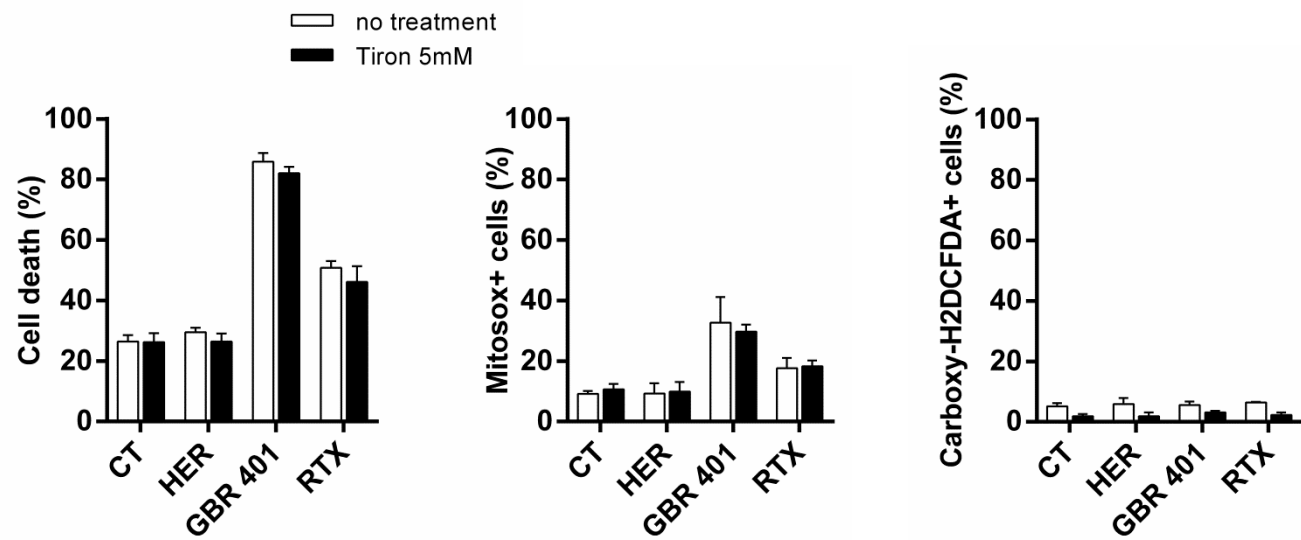

B

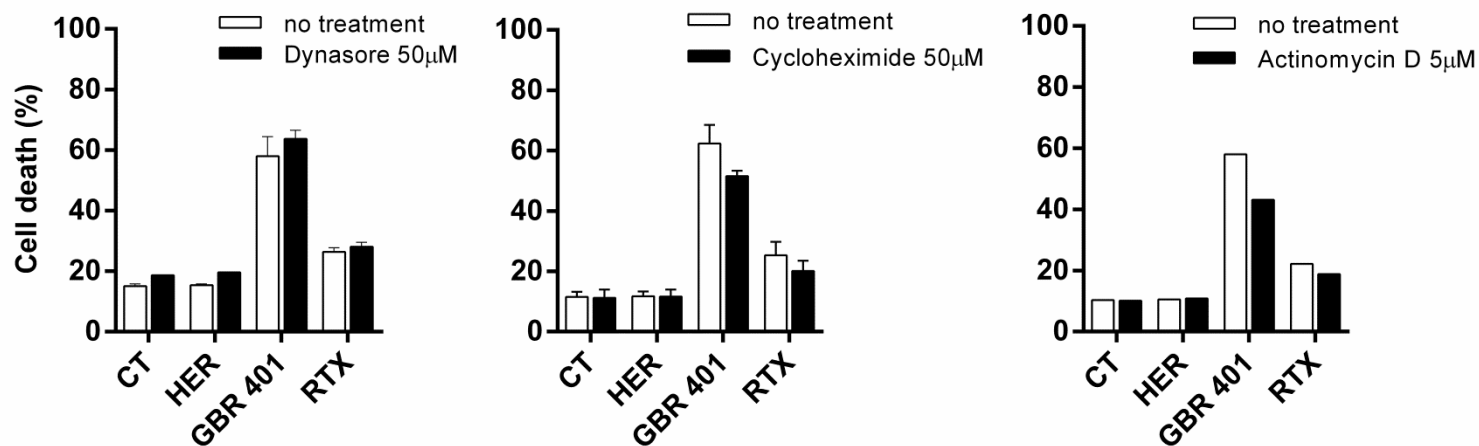

Figure S3

Supplement: Additional file 3: Figure S3 — GBR 401 cell death is not induced by ROS production, CD19 internalization or by mRNA and protein syntheses. A/ Raji cells were pre-incubated with the ROS scavenger Tiron and were treated for 2 hours with mAbs (1 μg/mL). Cell death (annexinV + 7AAD+/-), intracellular H2O2 (Carboxy- H2DCFDA) and mitochondrial superoxide (Mitosox) were assessed by flow cytometry. B/ Raji cells were pre-incubated with inhibitors of endocytosis (Dynasore), mRNA transcription (Actinomycin D) or protein synthesis (Cycloheximide) and were treated for 2 hours with mAbs (1 μg/mL). Cell death (annexinV + 7AAD+/-) was assessed by flow cytometry. [file 1756-8722-7-33-S3.pdf]
